# Feedback reporting of survey data to healthcare aides

**DOI:** 10.1186/1748-5908-7-89

**Published:** 2012-09-13

**Authors:** Alison M Hutchinson, Neha Batra-Garga, Lisa Cranley, Anne-Marie Bostrom, Greta Cummings, Peter Norton, Carole A Estabrooks

**Affiliations:** 1 School of Nursing and Midwifery, Deakin University, Melbourne, Victoria, Australia; 2 Cabrini-Deakin Centre for Nursing Research, Cabrini Health, Melbourne, Victoria, Australia; 3 Faculty of Nursing, University of Alberta, Edmonton, Alberta, Canada; 4 Department of Neurobiology, Division of Nursing, Care Sciences and Society, Karolinska Institutet, Huddinge, Sweden; 5 Department of Geriatric Medicine, Danderyd Hospital, Danderyd, Sweden; 6 Faculty of Medicine, University of Calgary, Calgary, Alberta, Canada

## Abstract

**Background:**

This project occurred during the course of the Translating Research in Elder Care (TREC) program of research. TREC is a multilevel and longitudinal research program being conducted in the three Canadian Prairie Provinces of Alberta, Saskatchewan, and Manitoba. The main purpose of TREC is to increase understanding about the role of organizational context in influencing knowledge use in residential long-term care settings. The purpose of this study was to evaluate healthcare aides’ (HCAs) perceptions of a one-page poster designed to feed back aggregated data (including demographic information and perceptions about influences on best practice) from the TREC survey they had recently completed.

**Methods:**

A convenience sample of 7 of the 15 nursing homes participating in the TREC research program in Alberta were invited to participate. Specific facility-level summary data were provided to each facility in the form of a one-page poster report. Two weeks following delivery of the report, a convenience sample of HCAs was surveyed using one-to-one structured interviews.

**Results:**

One hundred twenty-three HCAs responded to the evaluation survey. Overall, HCAs’ opinions about presentation of the feedback report and the understandability, usability, and usefulness of the content were positive. For each report, analysis of data and production and inspection of the report took up to one hour. Information sessions to introduce and explain the reports averaged 18 minutes. Two feedback reports (minimum) were supplied to each facility at a cost of CAN$2.39 per report, for printing and laminating.

**Conclusions:**

This study highlights not only the feasibility of producing understandable, usable, and useful feedback reports of survey data but also the value and importance of providing feedback to survey respondents. More broadly, the findings suggest that modest strategies may have a positive and desirable effect in participating sites.

## Background

Integration of research findings into practice is dependent, in part, upon researchers presenting and disseminating findings in an effective manner. Concern regarding communication of research findings is underpinned by evidence of the extent of the research–practice gap [1-11]. In healthcare the gap between what is known (research) and what is done (practice) is, in part, due to poor communication of research evidence to those responsible for care delivery [12]. Thus, in order to influence practice, the feedback of such data in a meaningful and useful manner is necessary. In particular, in the area of survey research, feedback of the findings is rarely undertaken. Further, limited evidence exists about the value of providing feedback of survey data [13]. A recent study of military personnel found that feedback of data and revelation of problematic areas or deficits was more likely to result in the survey being perceived as useful and, in turn, influenced respondents’ intentions to complete future surveys [14]. In a 2007 study of processes and formats for feedback of survey results to healthcare practices, researchers identified a useful and feasible feedback mechanism, which involved a feedback session comprising visual presentation of aggregated data in the form of dot plots [15].

From the knowledge-translation literature, evidence on the effectiveness of audit and feedback suggests that feedback of audit data has the potential to be effective in improving the practice of healthcare providers [16]. Healthcare aides (HCAs) are a unique group of healthcare providers. In the Canadian prairie provinces, they are unregulated workers who are either trained on the job, are students, or are graduates of short certificate programs. They constitute the largest direct provider group for care of nursing home residents [17]. Many HCAs, especially in urban areas, are immigrants, speak English as a second language, and have been largely neglected in terms of research to understand how they use knowledge in their practice [18,19]. Further, there is little research examining the effectiveness of knowledge-translation interventions in long-term care settings [19,20].

The present report describes the development and evaluation of a feedback intervention for HCAs. This project is designed to link directly with and complement the Canadian Institutes of Health Research-funded Translating Research in Elder Care (TREC) program of research, a multilevel and longitudinal research program being conducted in 36 nursing homes across the three Canadian prairie provinces: Alberta (n = 15), Manitoba (n = 8), and Saskatchewan (n = 13) [21-23]. The main purpose of TREC is to increase understanding about the role of organizational context in influencing knowledge use in long-term care settings. Within TREC there are two main inter-related projects and a series of pilot studies. The first of the main projects involves collection of quantitative data from several sources to explore and monitor the organizational context of nursing homes [22]. One of the main sources of data is an annual TREC survey administered to HCAs using computer-assisted personal interviews (CAPIs). The TREC survey is a suite of survey instruments that includes the Alberta Context Tool [24]. It is designed to assess organizational context and related factors believed to influence knowledge translation and the use of best practices. During year one of the TREC study, HCAs voiced a strong desire to receive feedback as the study progressed. In response to this request, the TREC research team initiated a feedback-reporting mechanism for HCAs in all facilities participating in the TREC research program.

The purpose of this study was to evaluate healthcare aides’ perceptions of a one-page poster designed to feed back aggregated data (including demographic information and perceptions about influences on best practices) from the TREC survey they had recently completed. The objectives of this project were to (1) evaluate the feasibility of providing feedback reports (process and format); (2) assess the understandability of the feedback reports (*i.e.*, whether the content could be comprehended); (3) assess the usability of the feedback reports—the preferred method of data presentation (*i.e.*, whether data were presented in a format that enabled its use); and (4) evaluate the usefulness/utility of the content of the feedback reports (*i.e.*, whether the variables selected for reporting were useful to inform behavior).

## Methods

The project had two distinct phases: (1) development of the report and (2) evaluation of the report.

### Development of the feedback report

The design of the feedback reports was guided by recommendations of a feasibility assessment undertaken in one long-term care facility in Alberta. That assessment was designed to determine the needs of HCAs with respect to the content and presentation of the feedback report and involved consultation with HCAs, care managers, and a site administrator to identify data of most interest and relevance for feedback purposes. The feasibility assessment indicated that a poster format, presenting the results from four survey variables in a graph form with minimal explanatory text and at a Flesch-Kincaid reading level of grade 8 or less, was optimal for this HCA population.

**Figure 1:**
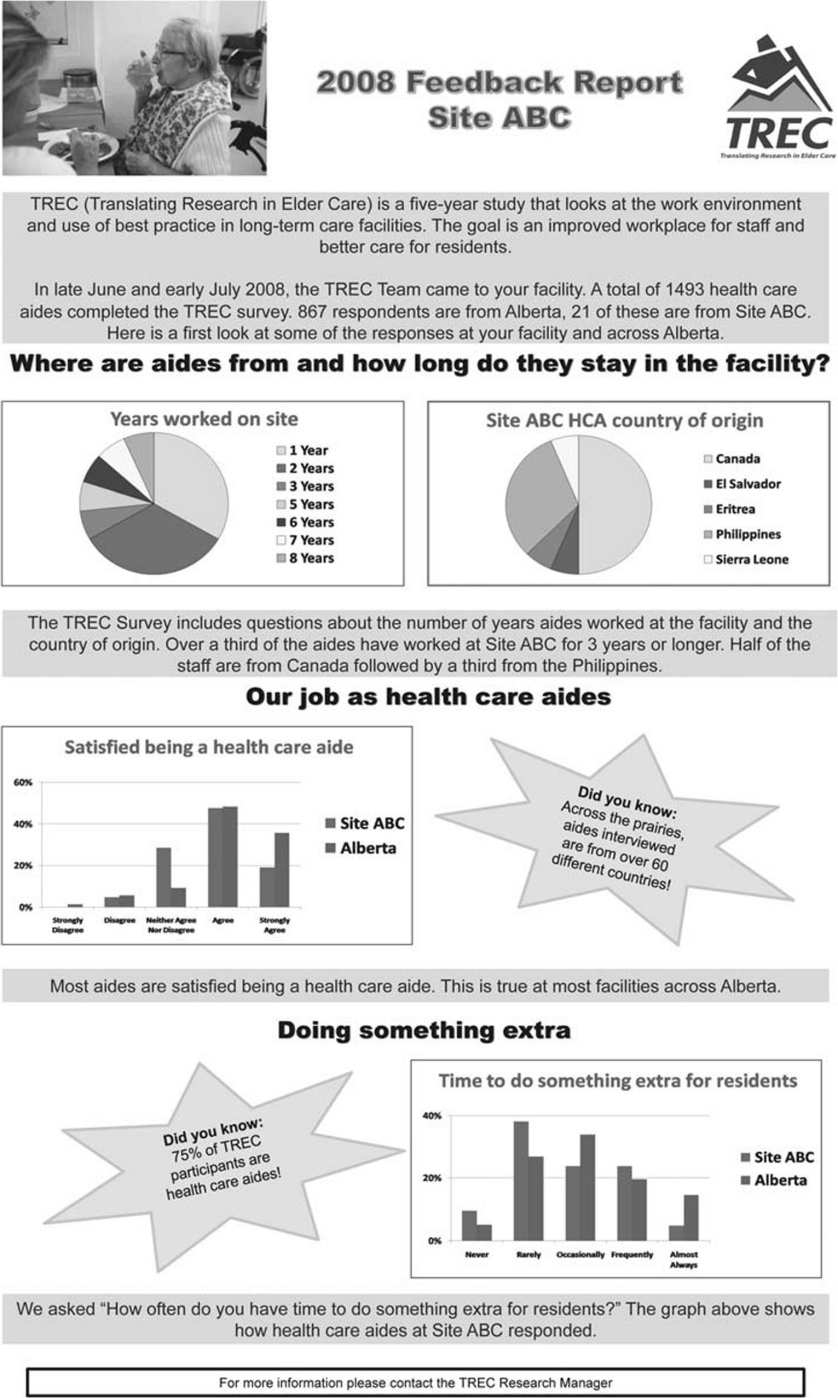
**Feedback poster report.** Example, in black and white, of a site feedback poster report.

Factors taken into consideration in planning the production and delivery of feedback reports included presentation format (verbal, paper, or electronic), frequency, content (which variables to report), and supporting processes (*e.g.*, information sessions). In developing the feedback reports, we adhered to general principles for presentation of data, including promotion of visual appeal (taking into consideration layout and use of white space, color, and illustrations) and making the content understandable (clear and concise presentation of information) and relevant to the HCAs. Guided by recommendations from the feasibility assessment, a one-page, legal-sized poster format was adopted for presentation of the results (Figure 1). The reports provided feedback to HCAs about select results from the year one TREC survey (which concluded in July 2009). Variables selected for inclusion in the feedback report were demographic characteristics, including country of origin, number of years worked at the facility, satisfaction with being a HCA (five-point Likert scale), and time to do something extra for the resident (five-point Likert scale). Facility-specific summary results were provided for each variable, along with comparative data reporting average provincial results from the TREC survey. Data were presented in graph form using pie charts and bar graphs. When developing the reports, careful consideration was given to reading level to ensure the reports were accessible to individuals of varying educational/reading levels. However, we found it difficult to achieve a Flesch-Kincaid grade reading level of 8 or less because some terms (for example, proper nouns such as country names) could not be simplified, and consequently, some segments of text exceeded this reading level.

### Evaluation of the feedback report

The evaluation of the feedback reports and reporting process involved (1) surveying (administered using one-to-one structured interviews) HCAs to elicit their opinions about the understandability, usability, and usefulness of the feedback reports and (2) collection of logistical data (such as time required to develop the reports, time required to deliver the reports, and cost of report production). While all facilities (n = 36) participating in the TREC research program received HCA feedback reports, a convenience sample of approximately 50% (n = 7) of the participating Alberta nursing homes (n = 15) was invited to participate in the evaluation of the HCA feedback reports.

### Distribution of the report

Once permission was granted by the facility administrator, the research assistants (who had administered the TREC survey approximately two weeks prior and were therefore well known in the facilities) arranged a suitable time with the unit managers in order to deliver the reports and provide a brief (15-minute maximum) information session for HCAs to explain the purpose and content and respond to questions about the report. Following each information session, the research assistants completed a brief evaluation form to document the number of HCAs in attendance and record the questions posed. At some of the larger facilities, more than one information session was required in order to access HCAs in each unit. In one province, an investigator also attended some feedback sessions. We recognized that delivery of feedback sessions required well-developed communications skills and careful preparation to ensure sensitive and appropriate responses to questions. To this end, standardized training (which involved in-person training delivered by the three Research Managers, with one training session held in each province), a training manual, and preparation time were provided to all research assistants.

## Data collection

### Evaluation survey data

Approximately two weeks following delivery of the feedback reports, HCAs who had previously completed a TREC survey in each of the participating facilities were invited to complete the evaluation survey. At each site included in the present study, 27 to 73 HCAs responded to the TREC survey, representing 39% to 96% of HCAs per site. We estimated that inclusion of 10 to 20 TREC survey respondents from these sites would be sufficient to achieve the objectives of this project. Across all 36 sites included in the TREC research, 9 to 164 HCAs per site responded to the TREC survey, representing, on average, 52% (range: 28%–96%) of HCAs at each site.

Our experience in TREC was that face-to-face structured interviews were the optimum method of survey data collection from HCAs. We found that this approach maximized data validity and minimized missing data. Hence, research assistants conducted short (5–10 minutes), one-to-one structured interviews with HCAs. The research assistants arranged a convenient time two weeks following delivery of the reports to conduct the structured interviews with HCAs. Written, informed consent was obtained prior to each interview, which was conducted in a quiet, private location in each facility. Structured questions elicited HCAs’ opinions about understandability of information included in the feedback reports; whether information was presented in a usable manner, that is, in a format that could inform practice or influence change; whether content of the report was relevant and useful to policy and practice; whether the report delivery process was appropriate in terms of timing and access to the report; and whether the accompanying information session was accessible and helpful. When a respondent identified deficits or weaknesses in the reports, the research assistants asked the HCA for suggestions or recommendations to improve the report. Examples of interview questions for each evaluation parameter are provided in Table 1.

**Table 1:** Examples of survey questions

**Feedback report evaluation parameters**	**Example**	**Response options**
*Presentation* of the report	Thinking about the appearance of the report, would you describe it as:	Very unattractive
		Somewhat unattractive
		Neither attractive nor unattractive
		Somewhat attractive
		Very attractive
*Understandability* of the report	Thinking about the language in the report, would you describe it as:	Very difficult to understand
		Somewhat difficult to understand
		Neither difficult nor easy to understand
		Somewhat easy to understand
		Easy to understand
*Usability* of the report	Thinking about use of the	Very unlikely
	information in the report, would you use it to	Somewhat unlikely
	influence the way you practice:	Neither likely nor unlikely
		Somewhat likely
		Very likely
*Usefulness* of the report	Thinking about the information in the report, how useful is this information to you in your role?	Not at all useful
		Slightly useful
		Somewhat useful
		Very useful
		Extremely useful
*Information session* content	Thinking about the information session, did you find the information that was presented:	Very difficult to understand
		Somewhat difficult to understand
		Neither difficult nor easy to understand
		Somewhat easy to understand
		Very easy to understand
Suggestions/recommendations	Do you have any suggestions for improving the appearance of the feedback report?	Open-ended questions

### Logistical data

Evaluation of the reporting process involved collection of logistical data on (i) time spent analyzing relevant TREC survey data and report production, (ii) time spent providing information sessions to introduce and explain the reports, (iii) the number of reports required by each facility, (iv) the most appropriate report topics, and (v) the cost of printing reports. Research assistants monitored the time invested for each activity and recorded these data in a spreadsheet on an ongoing basis as the study progressed.

### Ethical considerations

Human Research Ethics Board (HREB) approval for the conduct of this study was received from the University of Alberta, and permission of individual facility administrators was obtained to conduct the study in the respective facilities. In accordance with Tri-Council standards for research [25] and the HREB guidelines at the University of Alberta, informed consent was sought from HCAs prior to conducting the surveys.

### Data analysis

The evaluation survey data were double entered into a Statistical Package for Social Sciences (SPSS) version 17 (SPSS Inc., Chicago, IL, USA) database for analysis [26]. Descriptive statistics and cross tabulations were used to analyze evaluation survey data, and summary data described the logistical outcomes.

## Results

In total, 123 HCAs completed the evaluation survey (structured interview). On average, we surveyed 43% (range: 31%–70%) of the respondents to the TREC survey at each of the sites. The majority (94.3%) of respondents were female. On average, respondents had worked as a HCA for 9 years, had worked in the respective facility for 6.5 years, and 40% reported English as their second language (ESL) (Table 2). Demographic data for the TREC Alberta-only HCA sample and for the TREC Alberta, Manitoba, and Saskatchewan HCA sample are included in Table 2. These data demonstrate that the feedback survey respondents (n = 123) were demographically similar to the wider group of TREC survey respondents (Alberta only n = 864; Alberta, Manitoba, and Saskatchewan n = 1,489). Overall, healthcare aides’ opinions about the presentation of the feedback report and the understandability, usability, and usefulness of the content were positive (Table 3).

**Table 2:** Demographics

	**Present study (n = 123)**		**TREC Alberta (n = 864)**		**TREC Alberta, Manitoba, and Saskatchewan (n = 1,489)**	
	**N (%)**	**Mean (SD)**	**N (%)**	**Mean (SD)**	**N (%)**	**Mean (SD)**
Gender						
Males	7 **(**5.7)		50 **(**5.8)		92 **(**6.2)	
Females	116 **(**94.3)		803 **(**92.9)		1,386 **(**93.1)	
Age						
<20 years	3 **(**2.4)		8 **(**0.9)		13 **(**0.9)	
20–29 years	11 **(**9.0)		97 **(**11.2)		164 **(**11.0)	
30–39 years	35 **(**28.5)		191 **(**22.1)		312 **(**21.0)	
40–49 years	38 **(**30.9)		280 **(**32.4)		482 **(**32.4)	
50–59 years	28 **(**22.8)		230 **(**26.6)		400 **(**26.8)	
60–70 years	7 **(**5.7)		57 **(**6.6)		115 **(**7.7)	
>70 years	0 **(**0.0)		1 **(**1.0)		1 **(**1.0)	
Years worked as HCA		9.02 **(**6.9)		9.62 **(**8.7)		10.86 **(**8.9)
Years worked in facility		6.58 **(**5.9)		N/A		N/A
Education level						
High school	110 **(**89.4)		787 **(**91.1)		1,345 **(**90.3)	
HCA certificate	98 **(**79.7)		687 **(**79.5)		1,234 **(**82.9)	
Diploma or degree	60 **(**48.8)		450 **(**52.1)		658 **(**44.2)	
English as second language	48 **(**39.0)		449 **(**52.0)		660 **(**44.3)	

TREC = Translating Research in Elder Care; SD = standard deviation; HCA = healthcare aide; N/A = not available.

**Table 3:** Evaluation of feedback reports (N = 123)

	**N (%)**	**N (%)**	**N (%)**
**Presentation**			
Appearance	Unattractive	Neutral	Attractive
	9 **(**7.3)	5 **(**4.1)	109 **(**88.6)
Location on unit	Poor	Fair	Good
	19 **(**15.4)	20 **(**16.3)	84 **(**68.3)
**Understandability**			
Quantity of text	Too little	Neutral	Too much
	14 **(**11.4)	88 **(**71.5)	21 **(**17)
Language	Difficult	Neutral	Easy
	7 **(**5.7)	4 **(**3.3)	112 **(**91.1)
Graphics	Difficult	Neutral	Easy
	1 **(**0.8)	5 **(**4.1)	117 **(**95.2)
**Usability**			
To change practice	Unlikely	Neutral	Likely
	37 **(**30.1)	14 **(**11.4)	72 **(**58.6)
To change thinking about role	Unlikely	Neutral	Likely
	43 **(**35.0)	19 **(**15.4)	61 **(**49.6)
**Usefulness**	Not useful	Somewhat	Very
Usefulness of information	12 **(**9.8)	35 **(**28.5)	76 **(**61.8)
Usefulness in role	28 **(**22.8)	29 **(**23.6)	66 **(**53.7)

### Feedback reports

In total, 88.6% of respondents rated the feedback report as attractive. In terms of understandability of the report content, 71.5% of respondents were satisfied with the quantity of text provided, 91.1% of respondents indicated that the text was easy to understand, and 95.2% indicated that the graphics were easy to understand. With respect to the usability of the feedback, 58.6% of respondents said that they were likely to use the results to inform their practice, while 49.6% of respondents indicated that the findings were likely to change the way they thought about their role. When it came to usefulness of the data reported in the feedback report, 61.8% of respondents indicated that, in general, the information was very useful, with 53.7% indicating that the information was very useful in their role as HCAs.

Given that a large proportion of respondents reported having ESL, we tested for an association between language status and perceptions of the feedback report. After categorizing responses to the question about quantity of text into “too little,” “satisfactory,” or “too much,” the chi-square test for independence indicated a significant association between language status and perception of the quantity of text in the report (χ^2^(2, n = 123) = 14.97, *p* <.000). Specifically, fewer people with ESL were satisfied (approximately 50%) with the quantity of text in the poster when compared with the number of people with English as their first language who were satisfied (84%). However, a clear trend in the opinions of those with ESL about whether too much (as reported by 21% of ESL respondents) or too little text (as reported by 27% of ESL respondents) existed was not evident. Significant associations were also found between ESL and likelihood of the results to influence practice (χ^2^(2, n = 123) = 23.44, *p* <.000), likelihood of the results to influence thinking (χ^2^(2, n = 123) = 9.12, *p* = .011), perceptions of usefulness of the feedback in general (χ^2^(2, n = 123) = 22.17, *p* <.000), and usefulness of the feedback in their role (χ^2^(2, n = 123) = 24.70, *p* <.000). Specifically, a greater proportion of individuals with ESL indicated the results were likely to influence their practice and their thinking and reported that the information, in general and in their role, was useful.

### Information sessions

Attendance at the sessions was voluntary, and the number of individuals that attended was also subject to availability of HCAs on the days the sessions were scheduled. Of the 123 survey respondents, 52 (42.3%) had attended one of the information sessions at which the feedback reports were presented. Of these individuals, 61.5% reported that the information presented was either very useful or extremely useful. With regard to understandability, 40% reported that the information was easy to understand, and 48% reported that it was very easy to understand. We also analyzed the data for differences in perceptions of the feedback report based on attendance versus nonattendance at the information sessions. No statistically significant differences in perceptions were found between the two groups. Research assistants reported that, overall, the information sessions were well received by the HCAs and acted as a venue for HCAs to raise questions. Further, it was noted that HCAs expressed their gratitude towards the researchers for making the effort to return to the facility in order to feed back some of the study findings.

### Findings from logistical data

On average, up to one hour in total was spent by data analysts and investigators in analyzing the relevant TREC survey data for inclusion in the report and producing and inspecting the report for each facility. A minimum of two feedback reports (posters) per facility were supplied, and one additional poster was also supplied for every 50 beds in excess of 100 beds. Posters were produced in tabloid size (27.9 by 43.2 cm) and were laminated. The cost per poster for printing and lamination was CAN$2.39. In total, 7.35 hours were spent delivering the information sessions (n = 25) to facility staff, and the sessions averaged 18 minutes (range: 5–45). On average, four (range: 1–9) information sessions were conducted per facility.

## Discussion

Feedback represents an important part of the translation phase of the research process and has a range of potential benefits, including promoting the translation of evidence into practice and engagement of participants in future research. To enable feedback initiatives as part of the research process, the cost of providing feedback should be built into research budgets. In the following, our discussion will center on the consequences for practice and research and the feasibility of providing feedback to survey respondents.

### Consequences for practice of providing feedback to survey participants

Our broad purpose was to develop a knowledge-translation intervention that would assist in reducing the research–practice gap. Feedback of relevant data and presenting them in a format that is easily understood has the potential to facilitate ownership of the results and promote the implementation of research-based changes in practice. Further, other researchers report that engagement of and ownership by care providers is an important element in motivating practice change based on research evidence [27-29]. While our findings indicate that almost 60% of respondents felt the research findings were likely to inform their practice, approximately 62% reported that the findings were generally useful, and approximately 54% indicated that the findings were useful to them in their roles, these results could be a reflection of the types of variables selected for inclusion in the feedback report. In particular, the demographic data, while interesting, cannot be used to inform practice. The data for questions relating to satisfaction with role and the availability of time to do something extra for residents are more likely to stimulate discussion than inform the practice of HCAs. However, these data may have influenced managers’ and facility administrators’ approaches to resourcing and organization of care.

We were cognizant of the fact that we had to be careful and respectful about the type of information we were feeding back. In the feedback report development phase we consulted with HCAs, research assistants who were working closely with HCAs during the TREC study data collection, and facility managers. We asked them to consider and advise on which variables would be appropriate, of interest, and relevant to HCAs. Variable selection was therefore guided by their recommendations as well as our judgment regarding the potential sensitivity of the data. For example, we were careful to avoid reporting on variables such as leadership and staffing levels, which could have reflected negatively on a facility or could have been inflammatory or have caused controversy.

### Consequences for future research of providing feedback to survey respondents

HCAs expressed gratitude for the investigators’ efforts to provide feedback on the survey results. This undertaking was perceived as evidence that the investigators considered the HCAs important to the TREC research. This finding resonates with literature that has identified survey respondents’ dissatisfaction with not receiving feedback about study results, which, in turn, can prompt a sense of exploitation by researchers and potentially can lead to apathy about or disinterest in participating in research in the future [30,31]. Other research, consistent with the findings of our study, identified the importance of researchers feeding back the study findings in-person [13].

The feedback of research findings has implications for the wider research domain. The provision of feedback acknowledges respondents’ contribution to the research and, in the course of a study that has two or more data collection points, can be an important strategy to promote goodwill, future participant engagement, and trust. However, consideration needs to be given to the effects that such an activity may have on future data. Feedback may alter behavior and may, therefore, influence study findings, potentially confounding them. The purpose of the study will likely determine whether it is appropriate to feed back findings during the conduct of the research. In the case of the TREC program of research, an integrated approach, involving the knowledge users, was adopted. We recognized that our feedback could influence healthcare aides’ behavior, thereby influencing future results. However, we weighed this against the specific requests from HCAs for feedback and considered that failure to provide feedback could be detrimental to our research relationship and credibility and that this may negatively affect healthcare aides’ willingness to participate in future surveys in the TREC program of research. In the process of delivering feedback, careful consideration should also be given to the potential effects of feedback that might be perceived in a negative light. Such feedback should be delivered sensitively, and researchers should be cognizant that negative feedback may adversely affect future response rates.

### Feasibility of providing feedback to survey participants

We aimed to develop a cost-effective feedback mechanism that conveyed relevant data in a clear, useful, and usable format. The poster approach, which provided data in a visual display with the minimum amount of explanatory text required to describe the results, was well received. We found that once the initial feedback report poster template had been developed, the poster could be produced with ease, was inexpensive, and was a useful means of presenting information in an accessible manner. In all, investment of time and money to produce the posters and provide the information sessions was outweighed by the benefits of satisfying survey respondents through the delivery of feedback and enhancing the chances that they would respond to future surveys. A range of approaches could be adopted for the delivery of feedback. In deciding which approach to adopt, researchers should carefully consider the target audience and the context in which the findings are being delivered and then tailor their approach accordingly. The significant associations that we found between ESL and characteristics of the feedback point to the importance of considering the needs of audiences for whom English is not their first language when delivering feedback. Additionally, researchers should consider and monitor the resource requirements associated with delivery of the feedback [32].

### Limitations

There are some limitations associated with this study. In particular, the study was set in a small number of long-term care facilities in a single Canadian province. Therefore, while we achieved the number of responses for which we had aimed at the outset of the study, our data represent a limited number of HCAs working within a distinct setting. Given these constraints, we recommend caution when generalizing the study findings to other settings.

## Conclusion

This project was designed to explore feedback reporting for the communication of survey data to HCAs. The delivery of feedback in a poster format presenting information graphically and with the minimum amount of text required to explain the results was positively received in the HCA sample included in this study. The importance of providing feedback to research participants was reinforced by the responses of participants at the information sessions, who expressed their gratitude for the feedback and their keen interest in how the findings of the TREC study would be used to influence policy and practice.

## Competing interests

The authors declare that they have no competing interests.

## Authors’ contributions

AMH designed the study, provided leadership for the study, and lead the manuscript development. NBG provided valuable advice during the design of the study, managed the data collection phase, and contributed to the manuscript development. LC assisted in data collection and analysis. AMB contributed to the design of the feedback reports and manuscript development. GC is provincial site lead for the TREC research program in Alberta. PN is co-lead investigator for project one of the TREC research program. CAE is the principal investigator for the TREC research program and conceived the HCA feedback study. AMH, CAE, and GC participated in securing the funding for the project. CAE, GC, and PN provided commentary on the final submitted manuscript. All authors read and approved the final submitted manuscript.
